# Is Left Bundle Branch Pacing Feasible in Patients With Ventricular Septal Defect?

**DOI:** 10.1111/anec.70150

**Published:** 2026-01-20

**Authors:** Binbin Luo, Longfu Jiang, Lu Zhang, Jiabo Shen

**Affiliations:** ^1^ Department of Cardiology Ningbo No. 2 Hospital Ningbo China

**Keywords:** cardiac angiography, cardiac conduction system, electrophysiology, perimembranous ventricular septal defect, selective left bundle branch pacing

## Abstract

His bundle typically passes through the central fibrous body of the atrioventricular node and then enters the membranous portion of the interventricular septum, where it branches into the left and right bundle branches. The feasibility and safety of left bundle branch area pacing (LBBAP) in patients with perimembranous ventricular septal defect (pmVSD) have not been reported.

## Graphical abstract

This report presents two cases of left bundle branch pacing (LBBP) implantation in patients with perimembranous ventricular septal defect (pmVSD) and provides a novel approach to guide lead placement using both angiography and electroanatomic mapping, which ensures accuracy and reduces potential complications.

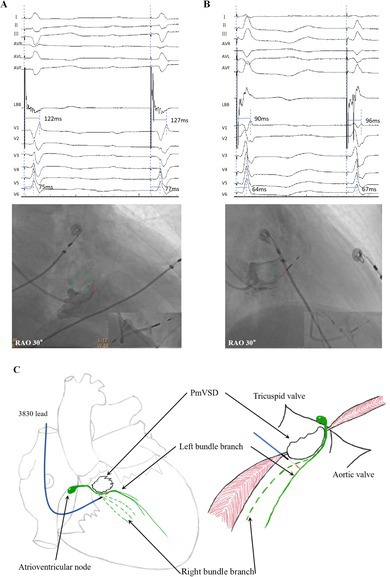

## 1 Introduction

Left bundle branch pacing (LBBP) has gained attention as a promising alternative to His bundle pacing in patients with left bundle branch block (LBBB), offering the advantages of stable thresholds and shorter QRS durations by pacing the left bundle branch beyond the block site (Feng et al. 2021; Vernooy et al. 2014). This technique allows for more physiological pacing and improved synchronization of ventricular contraction. In the context of congenital heart disease, ventricular septal defects (VSDs) represent one of the most common anomalies, with pmVSDs accounting for the vast majority of these cases. Because the ventricular septal defect leads to anatomical variations in the conduction bundle (Reemtsma and Copenhaver 1958; Latham and Anderson 1972; Titus et al. 1963), the feasibility of performing LBBP in these patients remains uncertain. This study aims to evaluate the feasibility and safety of LBBP in patients with VSDs, particularly those with pmVSDs, in order to explore its potential role in managing post‐procedural conduction disturbances.

## 2 Case Description

### 2.1 Case 1

**FIGURE 1:**
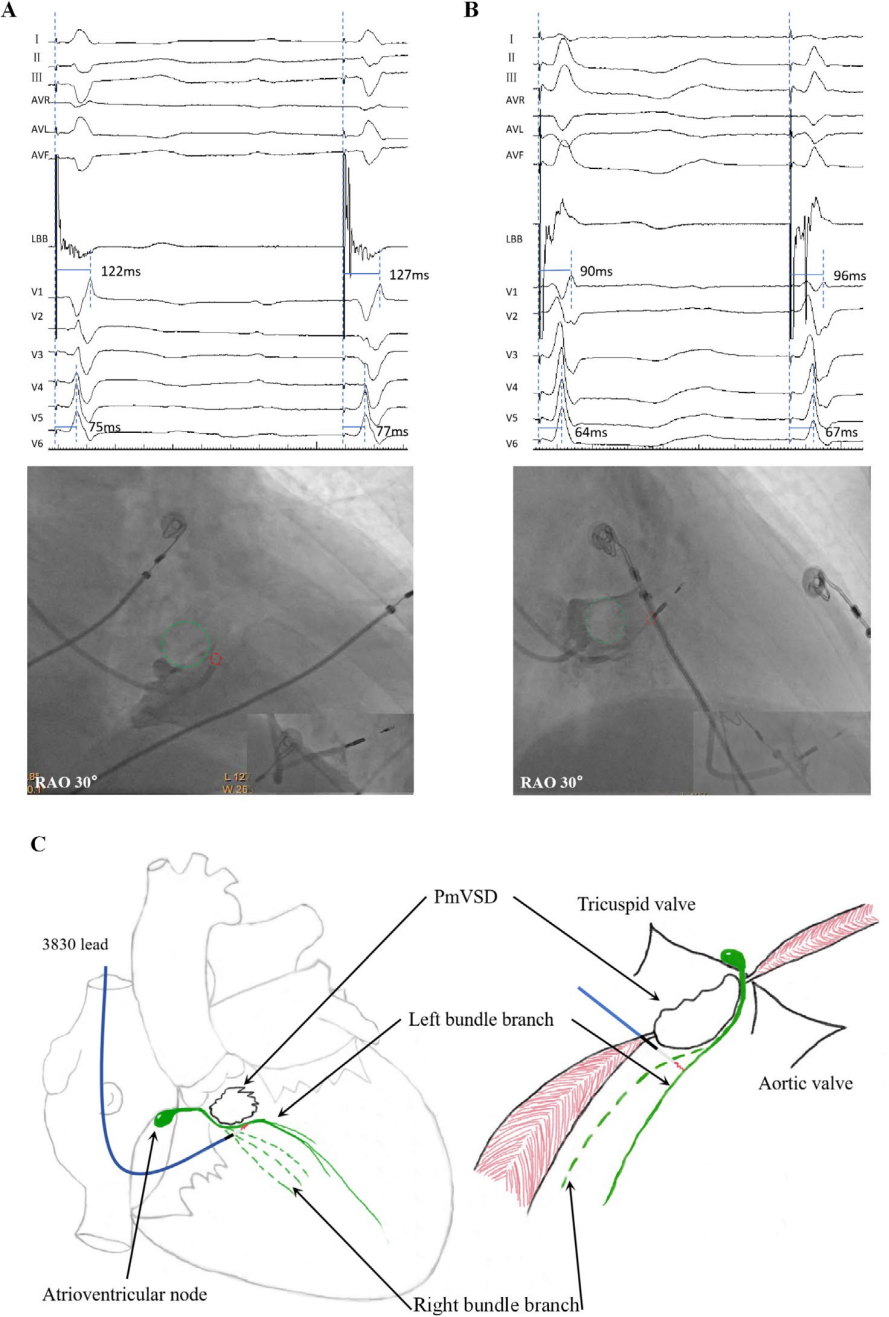
(A) Upon reaching the endpoint of implantation, a discrete EGM was observed in two consecutive heartbeats, approximate depth of 17 mm. (B) After the implantation of the electrode, a discrete EGM was observed within the adjacent heartbeats, approximate depth of 17 mm. In the RAO 30° right ventricular angiogram, the non‐contrast‐filled translucent area (Figure A/B, green circle) represents the contour of the perimembranous ventricular septal defect, caused by high‐velocity left‐to‐right shunting. Green circle:pmVSD; Red circle:Electrode insertion point. (C) Illustrates the relationship between electrodes at different angles reaching the left bundle branch and the pmVSD, which is located at the posterior‐inferior rim of the defect.

A 71‐year‐old male patient presented at the Emergency Department complaining of syncope. During admission, third‐degree atrioventricular block with complete right bundle branch block. Transthoracic echocardiogram showed an interruption of the basal echo continuity in the perimembranous portion of the interventricular septum, measuring 11 mm. The septum bulged towards the right ventricle, with an effective shunt opening measuring 4 mm. We calculated the ratio of pulmonary to systemic blood flow (Qp:Qs) to be 1.3:1. During right anterior oblique (RAO 30) right ventricular angiography, the non‐contrast‐filled translucent area (Figure 1A, green circle) represents the contour of the pmVSD, caused by high‐velocity left‐to‐right shunting. Medtronic 3830 SelectSecure lead (Medtronic Inc) was screwed into the lower‐right section of the radiolucent zone, reaching a depth of approximately 17 mm upon achieving the target left bundle branch area. Upon reaching the endpoint of implantation, a discrete EGM was observed in two consecutive heartbeats (Figure 1A).

### 2.2 Case 2

The second patient was a 66‐year‐old female patient who was admitted due to fatigue and dizziness. She was diagnosed with second‐degree, Mobitz type II atrioventricular block. Transthoracic echocardiography revealed a pmVSD at the 10 o'clock position in the short axis view. The defect showed an echo dropout of approximately 4 mm and protruded into the right ventricle in an aneurysm‐like fashion, measuring 16*13 mm. The Qp:Qs was measured to be 1.25:1 with a mean pulmonary pressure of 28 mmHg. In the right anterior oblique (RAO 30) view, right ventricular angiography revealed a circular radiolucent area near the tricuspid valve. The electrode was positioned at a relatively higher location (Figure 1B, red circle), also guided by the interventricular septal defect (Figure 1B, green circle). After S‐LBBP electrode implantation as the endpoint, a discrete EGM was observed between the adjacent heartbeats. The lead depth in the septum, which was 16 mm, was assessed using sheath angiography. The entire electrode implantation procedure can be referred to in the video (Video S1).

The patients consented to the publication of their clinical data and images for educational and research purposes.

## 3 Discussion

Ventricular septal defects are among the most prevalent congenital heart malformations, with approximately 70% occurring in the perimembranous region, located between the tricuspid and aortic valves. Transcatheter closure has become a widely used and effective treatment for pmVSDs (Minette and Sahn 2006). However, a significant complication that may arise during this procedure is atrioventricular block (AVB), which is believed to result from the proximity of the defect to the cardiac conduction system (Zhou et al. 2008). The pmVSDs have a fibrous continuity at its margin between the atrioventricular valves, forming the posteroinferior boundary of the defect. This region is where the atrioventricular conduction bundle emerges from the central fibrous body, transitioning into the subendocardial tissue. Given the location of the defect, both catheter manipulation and the pmVSDs occlusion device can potentially damage this conduction bundle, leading to AVB. The course of the atrioventricular bundle varies according to the type of VSD. In membranous defects, the bundle typically runs along the posterior edge of the defect, bifurcating at its inferior margin. In contrast, in muscular defects, the bundle bifurcates within the septum, with the left bundle descending to become closely associated with the anterior border of the defect (Latham and Anderson 1972; Titus et al. 1963). Kaur et al. employed 3D electroanatomic mapping (EAMS) to study the conduction system during transcatheter closure of pmVSDs. Their findings revealed that in all cases (100%), the conduction system was located posteroinferior to the defect, and in 67% of cases (10/15), it was situated away from the defect (Kaur et al. 2022). In our clinical experience with pmVSDs, the use of selective left bundle branch pacing can be guided by right ventricular angiography to assess the size and position of the VSD. Electroanatomic mapping, coupled with anatomical evaluation, helps in selecting an optimal lead insertion site, which is typically located inferior and posterior to the defect (Figure 1C). This approach ensures precise placement of the pacing lead at the terminal region of the left bundle branch, minimizing the risk of conduction system damage. In conclusion, the integration of selective left bundle branch pacing guided by right ventricular angiography, coupled with electroanatomic mapping, appears to be a promising approach for optimizing lead placement and minimizing the risk of AVB in these patients.

## 4 Conclusion

To the best of our knowledge, this is the first report of left bundle branch pacing in a patient with a ventricular septal defect. A small ventricular septal defect does not typically cause cardiac dysfunction. Although our experience is limited to this case, it suggests that using cardiac angiography to locate the ventricular septal defect and selecting the pacing lead insertion site based on bundle branch conduction characteristics may facilitate successful left bundle branch pacing.

## Author Contributions

Binbin Luo conceived the case, collected and interpreted the clinical data, performed the literature review, and drafted the initial manuscript. Lu Zhang contributed to data interpretation, assisted with figure preparation, and critically reviewed the manuscript for important intellectual content. Jiabo Shen participated in clinical management of the patient and contributed to manuscript revision. Longfu Jiang: Corresponding Author supervised the entire study, contributed to study design and data interpretation, critically revised the manuscript, and approved the final version for submission. All authors read and approved the final manuscript and agree to be accountable for all aspects of the work.

## Funding

The original study was supported by the Project of Ningbo Leading Medical & Health Discipline, China (2023Z191) and the Ningbo Major Research and Development Plan Project (2024Z235). The funders had no role in study design, data collection and analysis, decision to publish, or preparation of the manuscript.

## Conflicts of Interest

The authors declare no conflicts of interest.

## Supporting information


**Video S1:** (1) How to perform left bundle branch pacing in patients with Perimembranous ventricular septal defects. (2) The step‐by‐step sequence of the therapeutic modality that we presented. 00:00–00:19. (3) In the right anterior oblique 30° view, right ventricular angiography was performed via the 3830 sheath, revealing a radiolucent image of the ventricular septal defect. 00:20–02:29. (4) We screwed in the lead at the lower right corner of the defect under continuous monitoring until a discrete EGM was observed within the adjacent heartbeats.

## Data Availability

The data that supports the findings of this study are available in the Supporting Information of this article.
